# Bacterial Spectrum and Antibiotic Susceptibility Infection Patterns in the Lower Respiratory Tract: Insights From a Tertiary Care Hospital

**DOI:** 10.7759/cureus.108403

**Published:** 2026-05-06

**Authors:** Firoz B Shaik, Ravindra V Shinde, Satish R Patil

**Affiliations:** 1 Department of Microbiology, Krishna Institute of Medical Sciences, Krishna Vishwa Vidyapeeth (Deemed to be University), Karad, IND

**Keywords:** alternative therapies, antibiotic microbial resistance, antimicrobial stewardship, bacterial spectrum, laboratory diagnosis, lower respiratory tract infection, multidrug-resistant pathogens, rapid diagnostics, tertiary care centre

## Abstract

Lower respiratory tract infections (LRTIs) remain a significant global health concern with high mortality and morbidity rates. The increasing prevalence of multidrug-resistant (MDR) bacterial pathogens complicates the outcome of treatment. This review aims to highlight the bacterial spectrum linked with LRTIs and antibiotic susceptibility profiles through the synthesis of information in multiple studies conducted in tertiary care settings. The article examines epidemiological patterns, resistance mechanisms, and the implications of epidemiological patterns of clinical investigations conducted over the last decade through a narrative review. Moreover, it investigates how hospital infection control policies, factors affecting host immunity, and approaches to management affect the distribution of bacterial spectra and antibiotic patterns of sensitivity.

## Introduction and background

Lower respiratory tract infections (LRTIs) are inflammatory disorders affecting the airways below the larynx, which encompass the trachea, bronchi, bronchioles, and alveoli, and include conditions like pneumonia, bronchitis, and bronchiectasis [1]. They remain a major cause of global morbidity and mortality, especially affecting vulnerable groups including children, the elderly, and immunocompromised individuals.

The causative organisms vary based on the clinical context. In community-acquired infections, common pathogens include *Streptococcus pneumoniae, Haemophilus influenzae, *and *Moraxella catarrhalis*, as well as atypical agents like *Mycoplasma pneumoniae, Chlamydophila pneumoniae*, and *Legionella pneumophila* [2,3]. In contrast, hospital-acquired infections are more frequently linked to Gram-negative bacteria such as* Klebsiella pneumoniae, Pseudomonas aeruginosa, *and *Acinetobacter baumannii*, as well as *Staphylococcus aureus*, including methicillin-resistant *Staphylococcus aureus* (MRSA) [3,4]. Although *Escherichia coli *can sometimes be found in LRTIs, it is not a primary pathogen and is generally associated with specific high-risk or hospital-acquired settings.

Several factors increase susceptibility to LRTIs, such as chronic obstructive pulmonary disease (COPD), smoking, advanced age, immunosuppression, and previous antibiotic exposure. These factors are not direct causes of infection but are associated with an increased risk, especially for infections due to multidrug-resistant (MDR) organisms. The increasing occurrence of MDR pathogens, particularly in healthcare settings, has made the management of LRTIs even more challenging. This frequently leads to prolonged hospitalization and increased mortality. This highlights the importance of understanding local bacterial profiles and antimicrobial susceptibility patterns to inform suitable empirical treatment [3,4]. This review aims to comprehensively analyze the bacterial spectrum associated with LRTIs and their antimicrobial susceptibility patterns, with a focus on resistance trends and implications for clinical management.

Methodology

Research Strategy

The literature search primarily included studies published between 2014 and 2025, reflecting recent and relevant research on the bacterial spectrum and antimicrobial susceptibility patterns in LRTIs. One earlier study from 1998 was included due to its foundational relevance to antimicrobial resistance (AMR) mechanisms. Although no studies from 2021 and 2022 were included, all selected articles were carefully chosen based on their scientific relevance and contribution to the topic. Studies included in this review were those published in English that focused on the bacterial causes of LRTIs and reported antimicrobial susceptibility patterns. Studies conducted in hospital or tertiary care settings were primarily considered, and both original research articles and review articles with relevant microbiological data were included. Studies were excluded if they focused exclusively on viral, fungal, or non-bacterial infections, did not report antibiotic susceptibility data, or were case reports, editorials, or conference abstracts lacking sufficient detail. Duplicate studies were also excluded.

**Figure 1:**
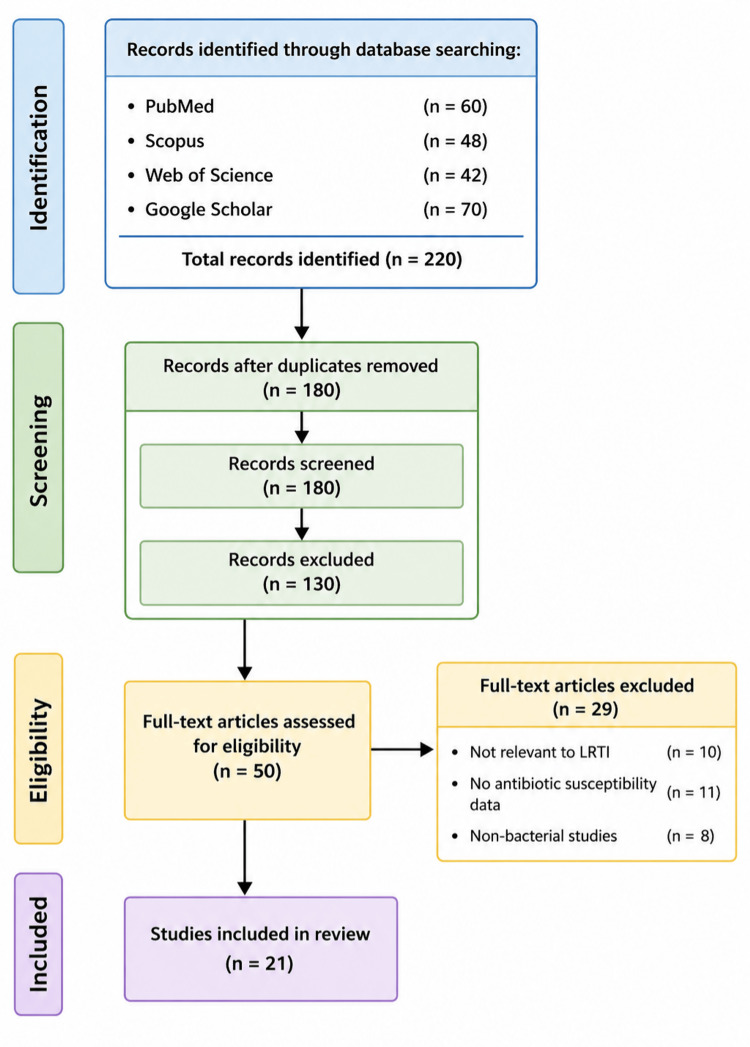
Article selection and screening process n: number of articles; LRTI: lower respiratory tract infection

Search strategies were tailored for each database to ensure comprehensive retrieval of relevant studies. For PubMed, the search included the terms ("lower respiratory tract infection" OR "LRTI") AND ("bacterial spectrum" OR "antibiotic susceptibility" OR "multidrug-resistant organisms"). For Scopus, the search strategy used was TITLE-ABS-KEY ("lower respiratory tract infection" AND "antibiotic resistance" AND "bacterial profile"). In Web of Science, the search was conducted using TS=("LRTI" AND "antimicrobial resistance" AND "bacterial pathogens"). For Google Scholar, the search terms used were "LRTI bacterial spectrum antibiotic susceptibility tertiary care". Boolean operators such as AND and OR were applied appropriately to refine and optimize the search results (Figure 1).

## Review

Epidemiology

The Global Burden of Disease (GBD) 2023 study reported about 2.5 million deaths and 98.7 million disability-adjusted life years of lower respiratory infections worldwide in 2023, with the highest disease burden observed among children below the age of five years and in persons aged 70 years and above. Although death rates among children under five have decreased by over a third since 2010, there are still significant geographical inequalities, especially in the areas of sub-Saharan Africa [5]. *Streptococcus pneumoniae* remains the most prevalent bacterial etiology of mortality in lower respiratory infection globally, followed by other important pathogens such as *Staphylococcus aureus* and* Klebsiella pneumoniae*. Emerging pathogens, including non-tuberculous mycobacteria and *Aspergillus* species, are increasingly contributing to the problem of lower respiratory infections, which further highlights the changing epidemiology. GBD 2023 is the most recent global assessment released as of 2025, and the estimates for later years are still not published [5].

Pathophysiology

There are many diseases that impair respiratory system function due to defects in the barrier mechanisms, hereditary abnormalities, or chronic inflammation. Although this is not an exhaustive list, the following conditions are typical examples of physiological consequences that follow the distortion of normal respiratory defence and clearance mechanisms [6].

Several common clinical conditions increase susceptibility to LRTIs by disrupting normal respiratory defense mechanisms. COPD is a major risk factor, as it causes reduced mucociliary clearance, excessive mucus production, and structural damage to the airways [7,8]. Other important risk factors include smoking, advanced age, diabetes mellitus, and immunosuppression, all of which weaken the host immune response and increase susceptibility to infection. Demographic factors, including age extremes (children and older adults), malnutrition, and inadequate socioeconomic conditions, significantly influence the development and severity of LRTIs. Moreover, pre-existing conditions like chronic lung disease, cardiovascular disease, and prior antibiotic exposure raise the likelihood of infections caused by MDR organisms and superinfections [6-11].

Asthma

Asthma is a chronic inflammatory respiratory condition marked by airway remodeling, increased bronchial hyperresponsiveness, and excessive mucus production. It impacts people of all ages and constitutes a major worldwide health issue. The airway mucosa experiences inflammation and edema, leading to bronchospasm episodes and airflow restriction, which are core pathophysiological features. Frequent triggers comprise allergens, respiratory infections, and environmental toxins. Bronchoconstriction occurs due to the contraction of smooth muscle in the airways, influenced by the interaction of airway epithelial cells, mast cells, smooth muscle cells, and parasympathetic nerves, clinically appearing as wheezing and dyspnea [6,7].

Cystic Fibrosis (CF)

CF is a recessive autosomal inherited disorder which is caused by the cystic fibrosis transmembrane conductance regulator (CFTR) gene mutation most commonly the DeltaF508 mutation. CFTR is a chloride channel which controls epithelial fluid. The effect of impaired CFTR activity leads to the over-reabsorption of sodium and water producing thick and dehydrated mucus that blocks airways and ducts. New technologies in the area of early diagnosis and targeted therapy have raised the average life expectancy up to about 40 years. CF is a multisystem disease affecting the lungs, pancreas, gastrointestinal tract, liver, and reproductive system, where pulmonary disease is the main cause of morbidity and mortality since it is involved in frequent infections and progressive lung destruction [6,10,11].

Ciliary Dyskinesia

Primary ciliary dyskinesia (PCD) is a condition that is genetically determined and is characterized by the impaired motility of respiratory cilia resulting in defective mucociliary clearance and the frequent occurrence of respiratory infections. There are clinical manifestations of chronic sinusitis, otitis media, bronchiectasis, and bronchi laterality defects, including situs inversus. Fertility may also be affected by ciliary dysfunction due to loss of sperm motility and dysfunction in the transport of the ovum. It is not diagnosed early enough and depends on ultrastructural ciliary and genetic analysis. The complex of chronic sinusitis, bronchiectasis, and situs inversus is known as the Kartagener syndrome [6,9,12]. The bacterial spectrum, resistance mechanisms, and empirical therapy in LRTIs are shown in Table 1.

**Table 1:** Bacterial spectrum, resistance mechanisms, and empirical therapy in LRTIs The main bacterial infections linked to LRTIs are listed in this table along with their resistance mechanisms, important genetic factors, and suggested empirical and alternative treatments. It emphasizes how multidrug-resistant organisms are becoming more common, especially among Gram-negative bacteria, which calls for focused antibiotic therapy. CAP: community-acquired pneumonia; HAP: hospital-acquired pneumonia; VAP: ventilator-associated pneumonia; MRSA: methicillin-resistant *Staphylococcus aureus*; PBP2a: altered penicillin-binding protein; ESBL: extended-spectrum β-lactamase; LRTIs: lower respiratory tract infections

Clinical condition	Pathogen	Resistance mechanism	First-line therapy	Alternative therapy
CAP	*Streptococcus pneumoniae* [5,12]	Altered penicillin-binding proteins; macrolide resistance [10,12]	Amoxicillin/ceftriaxone [10]	Levofloxacin [10]
	*Haemophilus influenzae* [2,3]	β-Lactamase production [12]	Amoxicillin-clavulanate [10]	Cefuroxime [10]
	*Staphylococcus aureus* (MRSA) [3,4]	PBP2a [12]	Vancomycin [10]	Linezolid [10]
	*Klebsiella pneumoniae* [3,4]	ESBL, carbapenemase production [10,12]	Ceftriaxone (if sensitive) [10]	Colistin, tigecycline, ceftazidime-avibactam [13]
HAP/VAP	*Pseudomonas aeruginosa* [3,4]	Efflux pumps, AmpC β-lactamase, porin loss [12]	Piperacillin-tazobactam [10]	Cefepime, meropenem [10]
	*Acinetobacter baumannii* [3,4]	Carbapenemase production, biofilm formation [13]	Carbapenems (if sensitive) [10]	Colistin, minocycline [13]
Lung abscess/severe infection	Mixed anaerobes and Gram-negative bacteria [12]	β-Lactamase production [12]	Piperacillin-tazobactam [10]	Clindamycin plus metronidazole [10]
Pediatric pneumonia	*Streptococcus pneumoniae*, *Haemophilus influenzae*, *Staphylococcus aureus* [5,12]	β-Lactam resistance; MRSA-associated resistance [12]	Amoxicillin/ampicillin [10]	Ceftriaxone; vancomycin (if MRSA is suspected) [10]
Acute bronchitis/bronchiolitis (secondary bacterial)	*Mycoplasma pneumoniae*, *Chlamydophila pneumoniae* [2,3]	Macrolide resistance [12]	Supportive care; macrolides if bacterial infection is suspected [10]	Doxycycline [10]

Pathogenesis

The development of LRTIs results from a complex interaction between invading pathogens and the immune defenses of the host. Infection usually starts when pathogens enter the lower respiratory tract through inhalation of aerosolized droplets, aspiration of oropharyngeal secretions, or, more rarely, hematogenous spread. Of these, microaspiration is viewed as the most prevalent pathway. In normal conditions, defense mechanisms like the mucociliary escalator, cough reflex, antimicrobial peptides, and alveolar macrophages prevent microbial invasion. Nevertheless, damage to these defenses caused by elements like smoking, viral infections, chronic illnesses, or mechanical ventilation promotes the colonization of pathogens. After colonization, pathogens adhere to respiratory epithelial cells, multiply, and evade host immune responses using virulence components like toxins and biofilm development. This triggers the activation of the host immune response, attracting inflammatory cells and prompting the release of cytokines, leading to alveolar inflammation, exudate formation, and compromised gas exchange, which are typical signs of pneumonia [4,11,12].

Intrinsic Immune Response in the Lung

The innate immune system of the lung offers fast initial protection against respiratory pathogens and therefore the outcome of the infection. Recent developments especially single-cell technologies have revealed the existence of a great deal of heterogeneity among innate immune cells showing immune responses to be highly context- and cell-specific. Discoveries made during the SARS-CoV-2 pandemic have also been used to explain immune activation, dysregulation, and host-pathogen interactions. A thorough knowledge of these pathways accounts for the creation of new treatment measures that will support in improving clearance of microbes without harming tissues as much [11].

Respiratory Tract Microbial Equilibrium

A dynamic microbial ecosystem exists in the respiratory tract to protect against the colonization of pathogens. The air moves in the nasal cavity through the nasopharynx and oropharynx to lower airways where the clearance of mucus and the mucociliary escalator help in the elimination of the inhaled particles and microbes. The colonization resistance of resident microbiota such as *Staphylococcus, Corynebacterium, Streptococcus, Moraxella, *and* Haemophilus* is a known factor to regulate immune functions and maintain epithelial barrier integrity. Healthy lungs are however not sterile; they carry with them a variety of microbial populations that inhibit the overgrowth of pathogens [12].

Pathogen Transition and Asymptomatic Colonization

There are many pathogens of LRTI including* Streptococcus pneumoniae, Haemophilus influenzae, Moraxella catarrhalis, *and *Staphylococcus aureus* that colonize the upper respiratory tract without symptoms. Viral infections have the capacity to alter the balance of microbes, thus enabling bacteria to invade. Formation of biofilm enhances colonization stability and host resistance. Even though colonization is not harmful as a rule, it allows the spread of microbes and, in some circumstances, may lead to the infection of the lower respiratory tract [12].

Treatment of LRTIs

The treatment of LRTIs, such as community-acquired pneumonia (CAP), hospital-acquired pneumonia (HAP), and ventilator-associated pneumonia (VAP), is guided by established clinical guidelines and depends on the severity of the illness, the presence of comorbid conditions, and the risk of MDR organisms. Empirical antimicrobial therapy is initiated promptly and later adjusted according to severity scores, renal function, allergy history, and microbiological results. For outpatients with CAP lacking comorbidities, first-line empirical treatment consists of amoxicillin, doxycycline, or a macrolide in regions with low resistance. Patients with comorbid conditions need a β-lactam in conjunction with a macrolide or doxycycline or a respiratory fluoroquinolone. Hospitalized CAP patients are managed using a β-lactam in conjunction with a macrolide or fluoroquinolone, whereas severe cases of CAP necessitate combination treatment. Coverage for MRSA or *Pseudomonas aeruginosa* is recommended only for patients with particular risk factors [10]. 

The management of HAP and VAP depends on mortality risk and the likelihood of MDR infection. Low-risk patients may be treated with monotherapy, whereas high-risk patients require combination therapy including MRSA coverage and antipseudomonal agents when clinically indicated. Carbapenems remain essential for the treatment of infections caused by extended-spectrum β-lactamase (ESBL)-producing organisms, while newer antimicrobial agents or colistin is reserved for highly drug-resistant organisms [10,13].

Emerging therapies and challenges

New agents have been developed in response to the increasing AMR trends including lefamulin, omadacycline, delafloxacin, cefiderocol, ceftolozane-tazobactam, and ceftazidime-avibactam, each having an improved activity profile compared to MDR pathogens [13]. 

Sulbactam/Durlobactam (SUL/DUR)

SUL/DUR is an amalgamation of sulbactam with a novel diazabicyclooctane β‑lactamase inhibitor that selects class A, C, and D enzymes. The agent has a strong activity in carbapenem-resistant *Acinetobacter baumannii* in which it exhibits a susceptibility of over 95% in vitro. In the phase IV ATTACK trial, SUL/DUR had non-inferior mortality rates and a much lower rate of nephrotoxicity relative to colistin. The profile of intrapulmonary penetration and a favorable combination are adequate in support of its application in HAP/VAP. SUL/DUR was approved by the FDA in 2023, but its high cost might limit its usage [13,14]. 

Cefepime/Enmetazobactam

Cefepime/enmetazobactam is an intravenous combination that is approved for HAP/VAP and is still active against ESBL-producing *Enterobacterales*, OXA-48, and AmpC beta-lactamases. It has been demonstrated that the drug exhibits effective lung penetration and high susceptibility rates as confirmed by clinical and pharmacokinetic studies making it an effective carbapenem-sparing option [13,15]. 

Aztreonam/Avibactam

Aztreonam/avibactam is active against metallo-beta-lactamase-producing Gram-negative pathogens such as MDR *Enterobacterales* and *Stenotrophomonas maltophilia*. Clinical trials were similar in their efficacy to regular therapy, and an acceptable tolerability profile was observed [13,15]. 

Implications for the clinical management of LRTIs in the era of AMR

Clinical management of LRTIs in the era of AMR has become increasingly complex and challenging. The problem of AMR has significantly changed the approach to the treatment of LRTIs which requires a multidisciplinary strategy. MDR extensively drug-resistant (XDR) and pan-drug-resistant (PDR) organisms have become the most common types of organisms leading to increased failure in treatment, increased hospitalization, and increased mortality. The most essential measures to fight AMR include improved diagnostics, improved antimicrobial therapy, and stewardship programs [16].

Timely and Efficient Diagnosis

Culture-based methods used in the past hinder the promptness of the proper therapy. Rapid molecular diagnostics, e.g., polymerase chain reaction (PCR), multiplex PCR panel, matrix-assisted laser desorption/ionization time-of-flight mass spectrometry (MALDI-TOF MS), as well as next-generation sequencing (NGS), help identify the resistance determinants early enough, guiding specific and timely treatment.

Artificial intelligence (AI) based on predictive models and biomarkers that incorporate procalcitonin (PCT) serves to improve the precision of empirical therapy further [17].

Optimization of targeted therapy and empirical therapy

Empirical Therapy Modifications

The local resistance patterns should be used in making therapeutic decisions. Increased resistance to β-lactam introduces respiratory fluoroquinolones or dual β-lactam therapy as an important part of the treatment of CAP [10]. Piperacillin-tazobactam, meropenem, or cefepime is empirically recommended as a treatment for HAP and VAP and used in critically ill patients as an adjunctive agent [18,19].

De-escalation and Stewardship

De-escalation is applied to de-selective pressure of resistance and toxicity in the pathogen after confirming that the pathogen is susceptible. Reduction in antibiotic courses (5-7 days) has also proved to be equally effective without damaging microbiome integrity [19].

Infection prevention and antimicrobial stewardship in LRTI management

Infection Control and Antimicrobial Oversight in LRTI Treatment

Preventing AMR in LRTIs requires not only enhancing therapeutic efficacy but also enforcing stringent infection prevention strategies alongside thorough antimicrobial stewardship initiatives focused on optimizing antimicrobial usage and minimizing selection pressure.

Contact Precautions and Isolation

Individuals infected with carbapenem-resistant organisms (CRO), which include carbapenem-resistant *Enterobacterales* (CRE), MRSA, and MDR *Acinetobacter* spp., necessitate rigorous infection control measures like contact isolation, cohorting, and compliance with hand hygiene standards. Utilizing suitable personal protective equipment (PPE) is crucial to avert nosocomial transmission in healthcare environments.

Protocols for Decolonization

Decolonization methods, such as intranasal mupirocin and chlorhexidine gluconate body washes, are successful measures in certain high-risk intensive care unit (ICU) patients to lower MRSA colonization and the risk of subsequent transmission [19].

Antimicrobial Management Approaches

Antimicrobial stewardship programs are essential in managing LRTI by encouraging the proper choice, dosage, administration, and length of antimicrobial treatments. Essential elements consist of applying empirical treatment guidelines informed by local antibiograms, promptly narrowing broad-spectrum antibiotics upon receiving culture results, and steering clear of unnecessary antibiotic administration in cases of viral or non-bacterial infections. Further strategies involve prospective audits with feedback, policies to restrict high-tier antibiotics, and ongoing monitoring of antimicrobial use and resistance patterns to inform institutional prescribing approaches [19].

Vaccination Strategies

Vaccination against *Haemophilus influenzae *type B (Hib),influenza, and *Streptococcus pneumoniae* (13-valent pneumococcal conjugate vaccine (PCV13), 23-valent polysaccharide vaccine (PPSV23)) prevents bacterial superinfections, thereby decreasing inappropriate antibiotic use [20,21].

Future prospects and gaps in the research on AMR management of LRTIs

Despite the progress made in the diagnostics, therapeutics, and stewardship of antimicrobial stewardship, there remain gaps in the research to manage the issue of AMR in LRTIs. The way forward in future research should be related to the discovery of new antimicrobials, precision medicine, improved surveillance, and the development of integrated international policies.

Advancing Novel Antimicrobials and Alternative Therapy

Next-generation antibiotics: It is critical to develop new antibiotics that utilize new different mechanisms such as bacterial ribosomal RNA, efflux pumps, and cellular division. Several agents including teixobactin and LpxC inhibitors have shown good activity and should be further tested in clinical studies. 

Non-antibiotic Strategies

Bacteriophage therapy, antimicrobial peptides, AMR determinants editing using CRISPR-Cas9, and host-directed immunotherapies are all options with promising initial results.

Improving Quick and Customized Diagnostics

Point-of-care testing: Real-time PCR, CRISPR-based diagnostics, and metagenomic sequencing are undeniably required to identify the pathogens at the earliest stage; nevertheless, their economic implementation into the everyday clinical workflow is not an easy task.

AI-Based Predictive Models

Machine learning algorithms have the ability to optimize empirical therapy choice by using resistance patterns, though validation and implementation studies need to be rigorous before such widespread use takes place.

Strengthening the Surveillance of Global AMR

Global surveillance networks: Increasing the real-time antibiogram databases and integrating the surveillance of genomic epidemiology can be used to increase the monitoring of resistance development at a global level.

Environmental and zoonotic AMR surveillance: Studies of the problem of antibiotic residues in the agricultural environment and the mechanisms of zoonotic infection are an important element of the One Health approach to stopping the spread of AMR. One should maximize stewardship and policy interventions.

Regulatory and Economic Policies

Regulatory and economic approaches are essential components in addressing AMR in LRTIs. Subscription-based antibiotic funding models and "market entry reward" mechanisms have been proposed to incentivize the development of novel antimicrobial agents while ensuring sustainable and cost-effective patient access. In addition, regulatory frameworks such as reimbursement policies linked to antimicrobial stewardship and restricted approval pathways for reserve antibiotics may help reduce inappropriate use and preserve antimicrobial efficacy. A coordinated global strategy involving healthcare institutions, regulatory authorities, and international surveillance systems is crucial to strengthen AMR monitoring, enhance antimicrobial stewardship practices, and improve the management of drug-resistant LRTI pathogens.

## Conclusions

LRTI is a global health burden, especially due to the increasing prevalence of AMR. The proliferation of MDR pathogens limits the choices of treatment, consequently raising the morbidity, mortality, and healthcare costs. Poor diagnostics encourages the improper administration of antibiotics, which further increases resistance. The development of molecular diagnostics, AI, and NGS is also essential to early diagnosis and accurate treatment. Addressing AMR requires policy changes, more funding for the development of antibiotics and other alternative therapies like bacteriophages and antimicrobial peptides, as well as improved surveillance in a One Health approach. The interaction between clinicians, researchers, and policymakers is also important in order to maintain and increase the effectiveness of antimicrobials.
